# Effects of lactulose on renal function and gut microbiota in adenine-induced chronic kidney disease rats

**DOI:** 10.1007/s10157-019-01727-4

**Published:** 2019-03-20

**Authors:** Miyu Sueyoshi, Masaki Fukunaga, Mizue Mei, Atsushi Nakajima, Gaku Tanaka, Takayo Murase, Yuki Narita, Sumio Hirata, Daisuke Kadowaki

**Affiliations:** 1 Department of Clinical Pharmacology, Faculty of Pharmaceutical Sciences, Kumamoto University, 5-1, Oe-Honmachi, Chuo-ku, Kumamoto, 862-0973 Japan; 2 Pharmaceuticals Research Laboratories, Sanwa Kagaku Kenkyusho Co., Ltd, Mie, Japan; 3 Center for Clinical Pharmaceutical Sciences, Faculty of Pharmaceutical Sciences, Kumamoto University, 5-1, Oe-Honmachi, Chuo-ku, Kumamoto, 862-0973 Japan; 4 Department of Clinical Pharmaceutics, Faculty of Pharmaceutical Sciences, Sojo University, 4-22-1 Ikeda, Nishi-ku, Kumamoto, 860-0082 Japan

**Keywords:** Lactulose, Renal function, CKD, Uremic toxin, Gut microbiota

## Abstract

**Background:**

Constipation is frequently observed in patients with chronic kidney disease (CKD). Lactulose is expected to improve the intestinal environment by stimulating bowel movements as a disaccharide laxative and prebiotic. We studied the effect of lactulose on renal function in adenine-induced CKD rats and monitored uremic toxins and gut microbiota.

**Methods:**

Wistar/ST male rats (10-week-old) were fed 0.75% adenine-containing diet for 3 weeks to induce CKD. Then, they were divided into three groups and fed as follows: control, normal diet; and 3.0- and 7.5-Lac, 3.0% and 7.5% lactulose-containing diets, respectively, for 4 weeks. Normal diet group was fed normal diet for 7 weeks. The rats were observed for parameters including renal function, uremic toxins, and gut microbiota.

**Results:**

The control group showed significantly higher serum creatinine (sCr) and blood urea nitrogen (BUN) 3 weeks after adenine feeding than at baseline, with a 8.5-fold increase in serum indoxyl sulfate (IS). After switching to 4 weeks of normal diet following adenine feeding, the sCr and BUN in control group remained high with a further increase in serum IS. In addition, tubulointerstitial fibrosis area was increased in control group. On the other hand, 3.0- and 7.5-Lac groups improved sCr and BUN levels, and suppressed tubulointerstitial fibrosis, suggesting preventing of CKD progression by lactulose. Lac groups also lowered level of serum IS and proportions of gut microbiota producing IS precursor.

**Conclusion:**

Lactulose modifies gut microbiota and ameliorates CKD progression by suppressing uremic toxin production.

## Introduction

The estimated prevalence of chronic kidney disease (CKD) is 8–16% worldwide, and Japan has also highest prevalence [1]. Further, concerns over the prevalence of CKD are increasing because its onset and progression have been attributed to aging and modern lifestyles in recent years [2]. Progression of CKD causes various pathologic conditions, including constipation due to dietary fiber insufficiency induced by restricted potassium intake and adverse reactions to potassium binders or other oral medications that induce constipation [3]. Bowel control is one of the key points from the perspective of maintaining or improving the patient’s quality of life (QOL).

Interestingly, a recent study in adenine-induced CKD mice reported that lubiprostone, a chloride channel activator used for chronic constipation, not only stimulates bowel movement but also improves the intestinal environment and suppresses the worsening of renal dysfunction by reducing the accumulation of uremic toxins [4].

On the other hand, lactulose reaches the large intestine in its unchanged form when administered orally. It helps retain intestinal water and electrolytes by increasing the osmotic pressure in the lower intestinal tract. Higher levels of intestinal water soften the intestinal content making it easier to move with looser stool and frequent bowel movements. Compared with sennosides and other irritant cathartics, lactulose as a laxative has less adverse reactions such as abdominal pain and is widely used as an osmotic laxative in other countries [5].

While lactulose is expected to further improve the intestinal environment by its dual function as a disaccharide laxative that stimulates bowel movement and as a prebiotic [6], few reports of its potential effect on the renal function are available. Here, we report our study in adenine-induced CKD rats evaluating the effects of lactulose on the renal function, uremic toxins, and gut microbiota.

## Materials and methods

### Reagents

Crystalline lactulose (purity ≥ 97%) was provided by Sanwa Kagaku Kenkyusho Co., Ltd. (Mie, Japan). Adenine was purchased from Wako Pure Chemical Industries, Ltd. (Osaka, Japan). Indoxyl sulfate (IS) potassium salt and 3-indoxyl sulfate-d4 (IS-d4) potassium salt were purchased from Sigma-Aldrich Corp. (St. Louis, MO, USA) and Toronto Research Chemicals (Toronto, Canada), respectively. Trimethylamine *N*-oxide (TMAO) dihydrate and trimethylamine *N*-oxide-d9 (TMAO-d9) were purchased from Tokyo Chemical Industry Co., Ltd. (Tokyo, Japan) and Cambridge Isotope Laboratories, Inc. (Tewksbury, MA, USA), respectively. *p*-Cresyl sulfate (PCS) was synthesized according to a published method [7].

### Experimental animals

The Wistar/ST male rats (9-week-old) purchased from Japan SLC, Inc. (Shizuoka, Japan). The animals were individually kept in an animal facility under controlled conditions, 23 ± 2 °C and relative humidity of 55 ± 10% under 12-h light/dark cycle with free access to feed (CE-2, CLEA Japan, Inc., Tokyo, Japan) and water. This study was approved by the institutional animal experiment committee at Kumamoto University and Sanwa Kagaku Kenkyusho Co., Ltd.

### Animal study

**Fig. 1:**
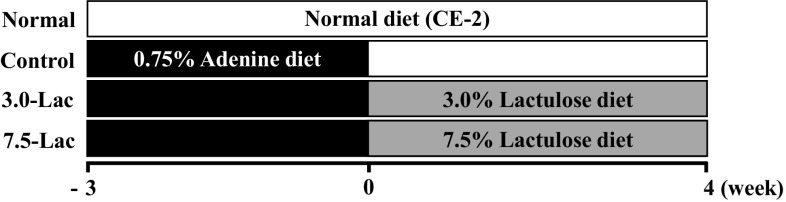
Experimental design

The study design is shown in Fig. 1. After 1-week acclimation, the rats were divided into two groups and fed either normal feed CE-2 (normal group, *n* = 12) or 0.75% adenine-containing CE-2 feed (*n* = 36) for 3 weeks. Then, the adenine-containing feed groups were divided into three groups (*n* = 12 each): control (fed CE-2 solid feed) and 3.0- and 7.5-Lac (fed CE-2 solid feed supplemented with lactulose at 3.0% and 7.5%, respectively) with comparable serum creatinine (sCr), BUN, serum IS, and body weight. The animals were observed for 4 weeks, and a normal group was fed normal CE-2 feed.

Blood samples were obtained under isoflurane anesthesia, from the subclavian vein before (week − 3) and 3 weeks after feeding the adenine-containing diet (week 0) and from the inferior vena cava on the last day of the normal or lactulose-containing diet (week 4). Blood samples were centrifuged at 3000×*g* for 10 min at 4 °C to separate the serum. sCr, BUN and other serum chemistry parameters were determined using an automatic analyzer (Hitachi 7180, Hitachi High-Technologies Corp., Tokyo, Japan). At 4 weeks, blood samples were collected in sampling tubes containing EDTA-2K and analyzed using a multi-parameter automatic hematology analyzer (XT-2000iV, SYSMEX Corp., Hyogo, Japan). The kidneys were weighed and sampled for histopathological examination at week 4.

### Measurement of oxidative stress markers

Serum concentrations of malondialdehyde (MDA) were determined using thiobarbituric acid reactive substances (TBARS) assay kit (Funakoshi Co., Ltd., Tokyo, Japan).

Serum concentrations of advanced oxidation protein products (AOPPs) were determined according to a method described by Witko-Sarsat et al. [8]. Serum samples were diluted fivefold in phosphate-buffered saline (PBS) and dispensed in 200-µL volumes in microplates, mixed with 20 µL acetic acid and 10 µL 1.16 M potassium iodide, and analyzed using a microplate reader (iMark, Bio-Rad Laboratories, Inc., Hercules, CA, USA.) to determine the absorbance at 340 nm.

### Measurement of antioxidant markers

Serum thiol content was determined using the 5,5′-dithiobis-(2-nitrobenzoic acid) (DTNB) method [9, 10]. Buffer A was prepared by mixing 100 mM potassium phosphate buffer (KPB, pH 7.0) and 1 mM diethylenetriamine pentaacetate (DTPA), and buffer B was prepared by mixing 10 mL buffer A and 2.0 mg DTNB. Serum samples were dispensed in 20 µL volumes in microplates and mixed with 100 µL buffer A (blank wells) or 100 µL buffer B (sample wells), incubated for 15 min, and analyzed by measuring the absorbance at 405 nm. The thiol content was calculated based on differences in absorbance readings between the sample and blank wells.

Serum reduced and oxidized glutathione (GSH and GSSG, respectively) were determined using a GSSG/GSH quantification kit (Dojindo Co., Ltd., Kumamoto, Japan) according to the supplier’s instructions.

### Measurement of IS, PCS, and TMAO

To measure IS and TMAO, serum samples were deproteinized with an ethanol solution containing an internal standard and analyzed using liquid chromatography–mass spectrometry (LC–MS) [11]. The internal standards were IS-d_4_, IS, and TMAO-d_9,_ TMAO. A high-performance LC (HPLC) system (Acquity UPLC^®^, Waters Corp., Milford, MA, USA) and a mass spectrometer (LTQ-Orbitrap, Thermo Fisher Scientific GmbH, Bremen, Germany) were used.

To measure PCS, serum samples were deproteinized with methanol and analyzed using HPLC [12]. An HPLC system equipped with an autosampler (AS-950, JASCO Corp., Tokyo, Japan), a pump (PU-980, JASCO Corp., Tokyo, Japan), and a fluorescence detector (RF-10A, Shimadzu Corp., Kyoto, Japan) was used.

### Histopathological examination of kidney tissue

Each kidney harvested at week 4 was fixed in 10% buffered formalin, paraffin embedded, cut into thin sections, and stained using Masson’s trichrome (MT). Stained sections were observed using a fluorescence microscope (BZ-X710, KEYENCE Corp., Osaka, Japan) and the tubulointerstitial fibrosis area was quantitated using a BZ-X analyzer.

### Quantitative real-time polymerase chain reaction (PCR) analysis

RNA was extracted using SYBER® premix Ex TaqTM II (TaKaRa Bio Inc. Japan). cDNA was synthesized using PC-818S (ASTEC CO. LTD., Japan) as a thermal cycler. The sequences of the primers used in this study were as follows: rat transforming growth factor (TGF)-β, forward 5′-TACAACAGCACCCGCGACCG-3′, reverse 5′-TGCGTTGTTGCGGTCCACCA-3′; rat GAPDH, forward 5′-CCTGGAGAAACCTGCCAAGTATG-3′, reverse 5′-TTGAAGTCACAGGAGACAACCTG-3′.

### Analysis of gut microbiota and short-chain fatty acid

Fecal samples were collected via forcible bowel movement by massaging the abdomen and anus of the rats at week − 3, 0, and 4. The fecal samples were subjected to terminal restriction fragment length polymorphism (T-RFLP) analysis of gut microbiota. Short-chain fatty acid concentrations were determined by analyzing the cecal content samples collected on the last day of the lactulose-containing diet using LC with post-column pH-buffered electroconductivity detection. Gut microbiota and short-chain fatty acid analyses were performed at TechnoSuruga Laboratory Co., Ltd. (Shizuoka, Japan).

### Statistical analysis

The data are expressed as mean ± SD and compared using an analysis of variance (ANOVA) with Tukey–Kramer test and differences. *p* < 0.05 was considered statistically significant. Serum IS and sCr or BUN were analyzed for correlation using single regression analysis.

## Result

### Body weight, food consumption, and hematology

**Fig. 2:**
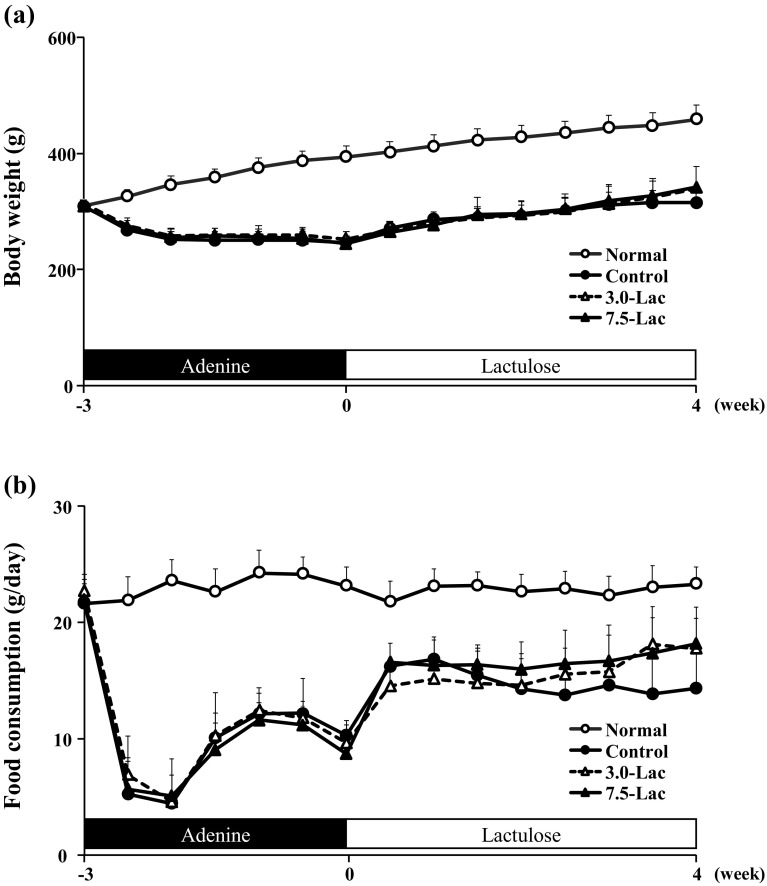
Effects of lactulose on body weight and food consumption of adenine-induced chronic kidney disease (CKD) rats. Changes of body weight (**a**) and food consumption (**b**) during adenine feeding and normal or lactulose feeding period in adenine-induced CKD rats. Values are expressed as the mean ± SD; *n* = 10–12/group

Time-course profiles of body weight and food consumption at week − 3, 0, and 4 are shown in Fig. 2. Hematology and serum chemistry findings at week 4 are summarized in Table 1. Three animals died during the study. Two died at week 3 and week 4 in the control group, and the other at week 4 in the 3.0-Lac group. The body weight of the control group decreased rapidly after adenine feeding, followed by a stable level for the rest of adenine feeding. After switching to normal diet following 3 weeks of adenine feeding, the body weight of control group gradually increased. Both of Lac groups exhibited a profile similar to that of the control group. The food consumption showed a sharp decrease after start of adenine feeding, followed by a recovery from 1 week later. The control group showed a further increase in food consumption after switching to a normal diet, followed by a gradual decrease until the study ended. The Lac groups showed a further increase in food consumption after switching to the lactulose-containing diet, followed by a gradual increase until the study ended. Additionally, no constipation and no diarrhea were observed in any groups during the study period. Hematologically, the red blood cell count, hemoglobin, and hematocrit levels of the control group markedly decreased compared with the normal group, while the Lac group parameters improved relative to the control in a dose-dependent manner. Moreover, the hypocalcemia that observed in control group improved at the same level as normal group by Lac. The white blood cell count was increased by the lactulose diet.

**Table 1:** Effects of lactulose on hematology and serum chemistry findings of adenine-induced chronic kidney disease (CKD) rats at week 4

	Normal	CKD		
		Control	3.0 Lac	7.5 Lac
RBC (10^4^/µL)	907.8 ± 31.0	551.8 ± 78.9**	617.2 ± 57.7**^, #^	661.5 ± 47.7**^, ##^
WBC (10^2^/µL)	107.4 ± 18.6	119.4 ± 21.3	166.8 ± 38.8**^, ##^	171.3 ± 17.7**^, ##^
HGB (g/dL)	16.0 ± 0.3	10.0 ± 1.4**	10.9 ± 1.0**	11.8 ± 0.9**^, ##^
HCT (%)	45.8 ± 0.7	30.4 ± 4.0**	33.1 ± 3.7**	35.6 ± 3.0^**, ##^
RET (10^4^/µL)	30.9 ± 2.2	33.8 ± 9.1**	40.0 ± 7.9**	40.9 ± 7.2**
Na	140.0 ± 1.4	139.4 ± 3.0	138.9 ± 2.1	140.1 ± 2.0
K	4.7 ± 0.2	6.1 ± 0.4**	6.3 ± 0.9**	5.7 ± 0.4**
Cl	100.6 ± 1.6	98.6 ± 4.1	99.7 ± 2.1	100.9 ± 2.2
Ca (mg/dL)	9.7 ± 0.3	8.7 ± 1.4*	9.7 ± 0.4^#^	9.7 ± 0.5^#^
IP (mEq/dL)	6.2 ± 0.4	8.9 ± 1.7**	7.7 ± 0.7**	7.8 ± 1.1**
Albumin (g/dL)	2.5 ± 0.1	2.2 ± 0.1**	2.3 ± 0.2**	2.3 ± 0.1*
Globulin (g/dL)	2.9 ± 0.2	2.7 ± 0.1*	2.7 ± 0.2**	2.8 ± 0.1
Albumin/globulin	0.9 ± 0.04	0.8 ± 0.04*	0.8 ± 0.05	0.8 ± 0.04
Total protein (g/dL)	5.4 ± 0.2	4.9 ± 0.3**	4.9 ± 0.4**	5.1 ± 0.1*
AST (IU/L)	87.5 ± 12.0	63.6 ± 7.4**	79.1 ± 14.9	77.7 ± 23.7
ALT (IU/L)	57.7 ± 5.9	52.3 ± 7.5	47.4 ± 9.9	53.6 ± 14.1
LDH (IU/L)	410.7 ± 131.5	166.7 ± 66.1	408.0 ± 372.2	337.4 ± 318.8
ALP (IU/L)	829.6 ± 152.5	883.3 ± 142.6	900.1 ± 105.2	916.1 ± 100.6
Total bilirubin (mg/dL)	0.003 ± 0.007	0.001 ± 0.003	0.004 ± 0.008	0.001 ± 0.003
Phospholipids (mg/dL)	124.9 ± 15.9	200.0 ± 31.4**	187.5 ± 21.1**	185.7 ± 18.4**
TG (mg/dL)	59.3 ± 26.6	96.0 ± 46.8	79.5 ± 34.9	57.8 ± 15.8^#^
Total cholesterol (mg/dL)	71.5 ± 11.0	133.2 ± 20.3**	124.5 ± 19.8**	124.8 ± 15.0**
CK (IU/L)	325.9 ± 148.1	152.1 ± 53.9	292.2 ± 215.8	245.8 ± 169.3
Glucose (mg/dL)	145.3 ± 19.1	130.0 ± 14.1	133.6 ± 15.1	133.2 ± 12.5

Values are expressed as the mean ± SD; *n* = 10–12/group

*p < 0.05 and **p < 0.01 vs. normal group, and ^#^*p* < 0.05 and ^##^*p* < 0.01 vs. control group

*RBC* red blood cell, *WBC* white blood cell, *HGB* hemoglobin, *HCT* hematocrit, *RET* reticulocyte, *IP* inorganic phosphorus, *AST* aspartate aminotransferase, *ALT* alanine aminotransferase, *LDH* lactate dehydrogenase, *ALP* alkaline phosphatase, *TG* triglycerides, *CK* creatine kinase

### Renal function

**Fig. 3:**
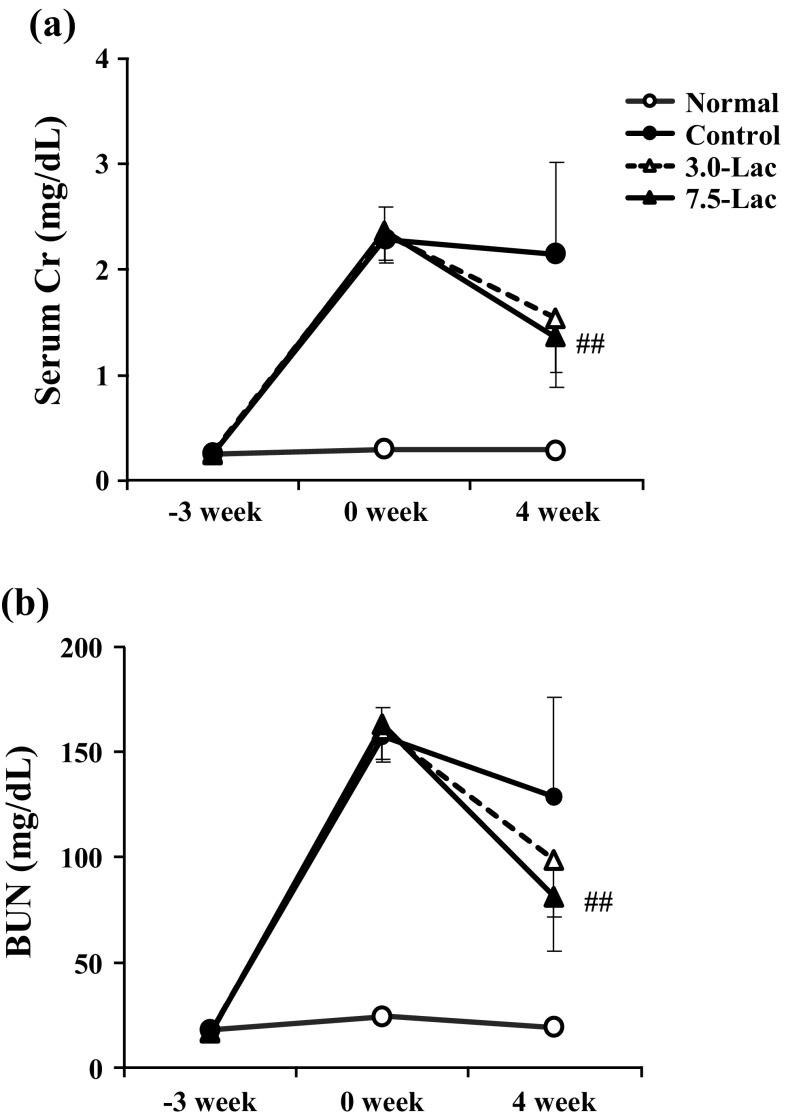
Effects of lactulose on renal functions of adenine-induced chronic kidney disease (CKD) rats. Serum creatinine (**a**) and blood urea nitrogen (BUN, **b**) in adenine-induced CKD rats. Values are expressed as the mean ± SD; *n* = 10–12/group. ^##^*p* < 0.01 vs. control group at week 4

Time-course profiles of sCr and BUN at week − 3, 0, and 4 are shown in Fig. 3a, b, respectively. Three weeks of the adenine-containing diet caused significant increase in sCr and BUN levels. The control group continued to have high sCr and BUN levels (2.15 and 128.5 mg/dL at week 4, respectively) during a period from week 0 to 4. The 3.0- and 7.5-Lac groups showed improved sCr level (1.54 and 1.36 mg/dL, respectively) at week 4 compared with levels before lactulose diet (week 0). The 7.5-Lac group showed significantly lower levels than the control group did (*p* < 0.01). The BUN level of the 7.5-Lac group was also significantly lower than that of the control group (81.1 mg/dL, *p* < 0.01).

### Uremic toxins

**Fig. 4:**
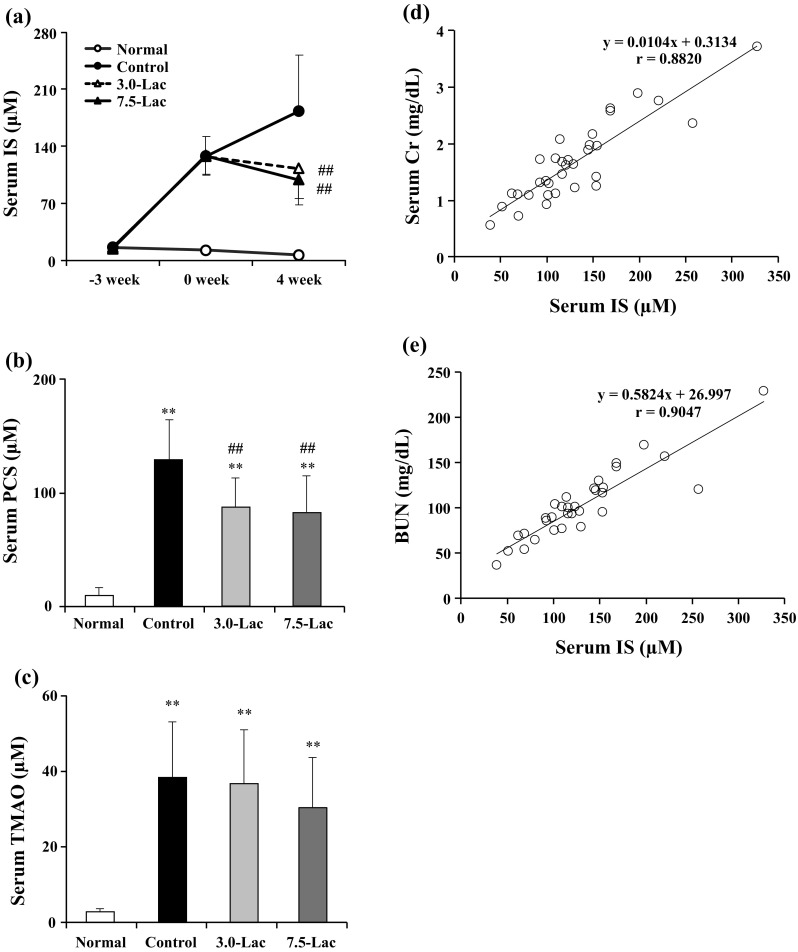
Effects of lactulose on uremic toxins of adenine-induced chronic kidney disease (CKD) rats. Serum concentration of indoxyl sulfate (IS. **a**), *p*-cresyl sulfate (PCS, **b**) and trimethylamine *N*-oxide (TMAO, **c**) levels in adenine-induced CKD rats. Relationship between serum IS and serum creatinine (Cr, **d**) or blood urea nitrogen (BUN) levels (**e**) in adenine-induced CKD rats; *n* = 33. Values are expressed as the mean ± SD; *n* = 10–12/group. **p < 0.01 vs. normal group. ^##^*p* < 0.01 vs. control group

The time-course profiles of serum IS at week − 3, 0, and 4 are shown in Fig. 4a. The control group serum IS level at week 0 was higher by approximately 8.5-fold than the level at week − 3. The control group showed further increased serum IS levels at week 4 compared with the level at week 0. On the other hand, both of Lac groups showed decreased serum IS levels compared with the level before the lactulose diet. Both of Lac groups showed significantly lower levels than the control group did (*p* < 0.01). Single regression analyses of serum IS and sCr or BUN levels at week 4 showed high correlations for both analyses (*r* = 0.88, *r* = 0.90, respectively; Fig. 4d, e). The control group showed markedly increased serum PCS at week 4 compared with the normal group, whereas in the Lac groups the increase was suppressed relative to the control group (Fig. 4b). In addition, the 7.5-Lac group showed a slightly lower serum TMAO than that of the control group, which increased at week 4 (Fig. 4c).

### Oxidative stress

**Fig. 5:**
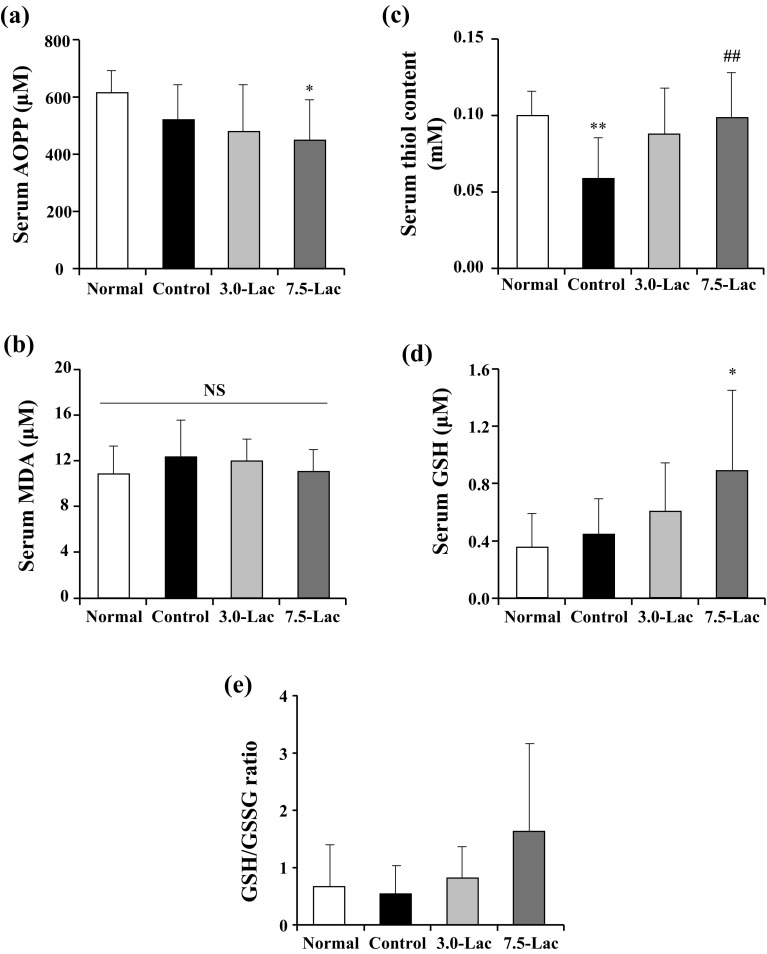
Effects of lactulose on oxidative stress markers (**a, b**) or antioxidant capacity (**c**–**e**) in adenine-induced chronic kidney disease (CKD) rats. Serum advanced oxidation protein products. (AOPPs, **a**), serum malondialdehyde (MDA, **b**), serum thiol continent (**c**), serum reduced glutathione (GSH, **d**) and GSH/oxidized glutathione (GSSG) ratio (**e**). Values are expressed as mean ± SD; *n* = 9–12/group. *p < 0.05 and **p < 0.01 vs. normal group and ^##^*p* < 0.01 vs. control group

The serum AOPPs level, as an oxidative stress marker at week 4, significantly decreased in the 7.5-Lac group compared with that in the normal group (Fig. 5a). Serum MDA levels were comparable across the four groups (Fig. 5b). Serum thiol content, determined as a measure of antioxidant capacity, was decreased in the control group and significantly improved in the 7.5-Lac group (Fig. 5c). Serum GSH levels were significantly higher in the 7.5-Lac group than in the normal group, and the GSH/GSSG ratio exhibited a similar trend (Fig. 5d, e).

### Kidney weight and morphological changes

**Fig. 6:**
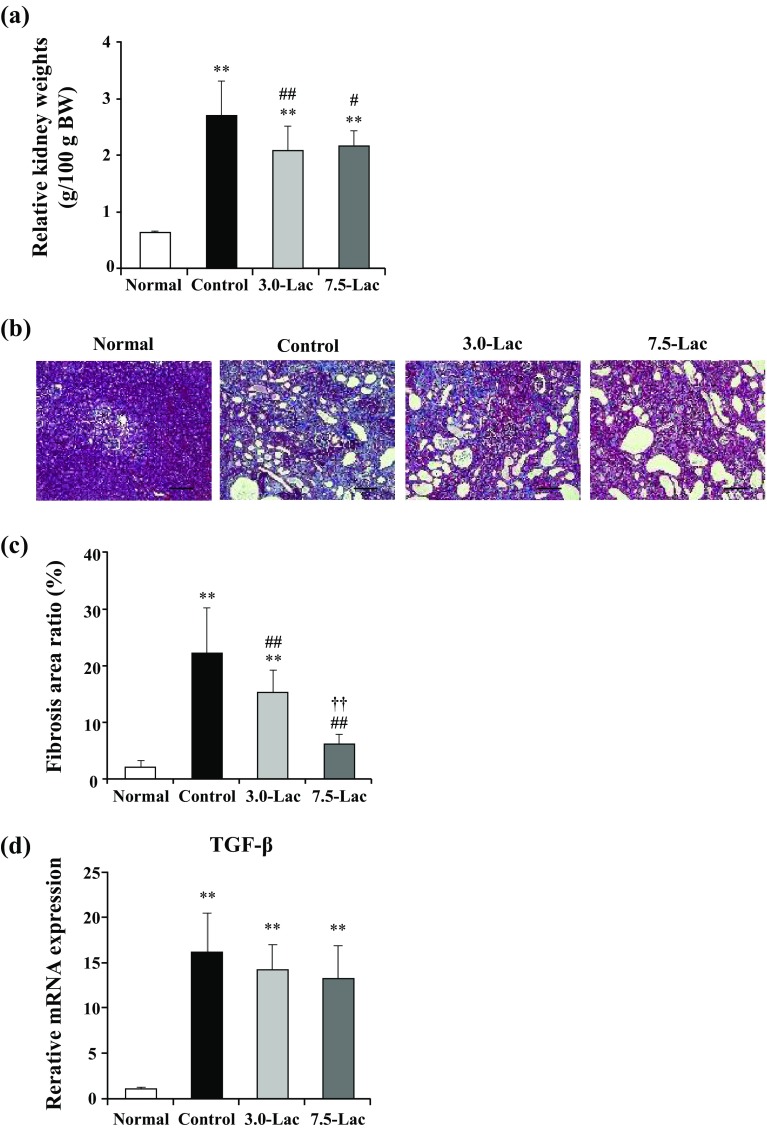
Effects of lactulose on relative kidney weights (**a**) and renal fibrosis (**b–d**) in adenine-induced chronic kidney disease (CKD) rats. Representative micrographs showing Masson’s trichrome (MT) staining (**b**). Scale bar, 200 µm. Fibrosis was digitally quantified and is shown as percentage of blue area of MT stain in kidney section (**c**). TGF-β mRNA expression was examined with quantitative PCR (**d**). The expression levels were normalized to the levels in the kidney from the normal rats. Values are expressed as mean ± SD; *n* = 10–12/group. **p < 0.01 vs. normal group, ^#^*p* < 0.05 and ^##^*p* < 0.01 vs. control group, and ^††^*p* < 0.01 vs. 3.0-Lac group

Data of the relative kidney weight per unit body weight at week 4 are shown in Fig. 6a. Representative micrographs of MT-stained kidney tissues at week 4 are presented for each group in Fig. 6b and the data of the relative tubulointerstitial fibrosis area are shown in Fig. 6c.

The relative kidney weight and relative fibrosis area at week 4 remarkably increased compared with that in the normal group. These increases were significantly suppressed in the Lac groups. Quantitative real-time PCR indicated a slight decrease of TGF-β, but not significantly (Fig. 6d).

### Gut microbiota and short-chain fatty acids

**Fig. 7:**
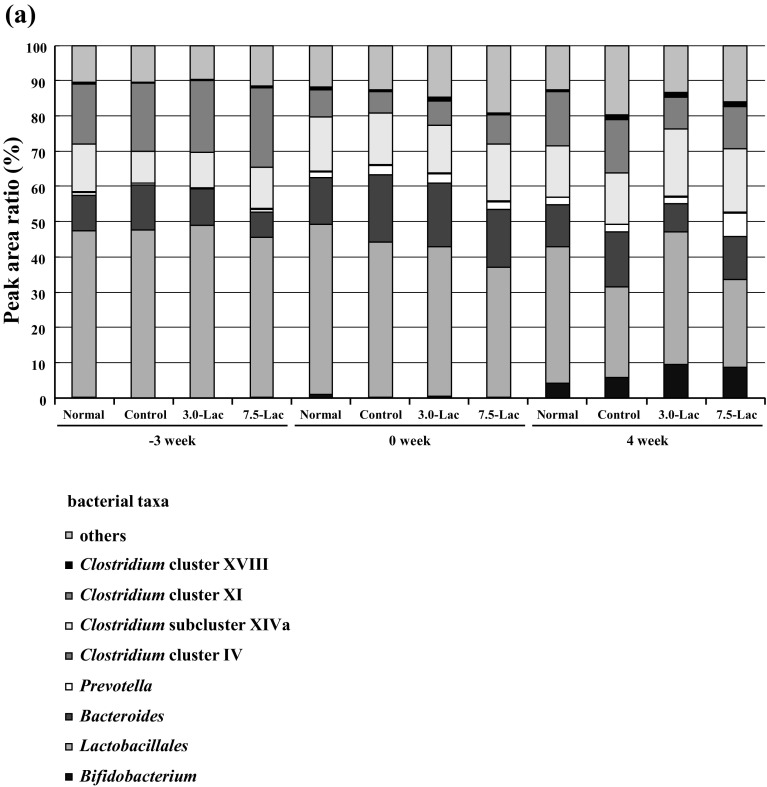

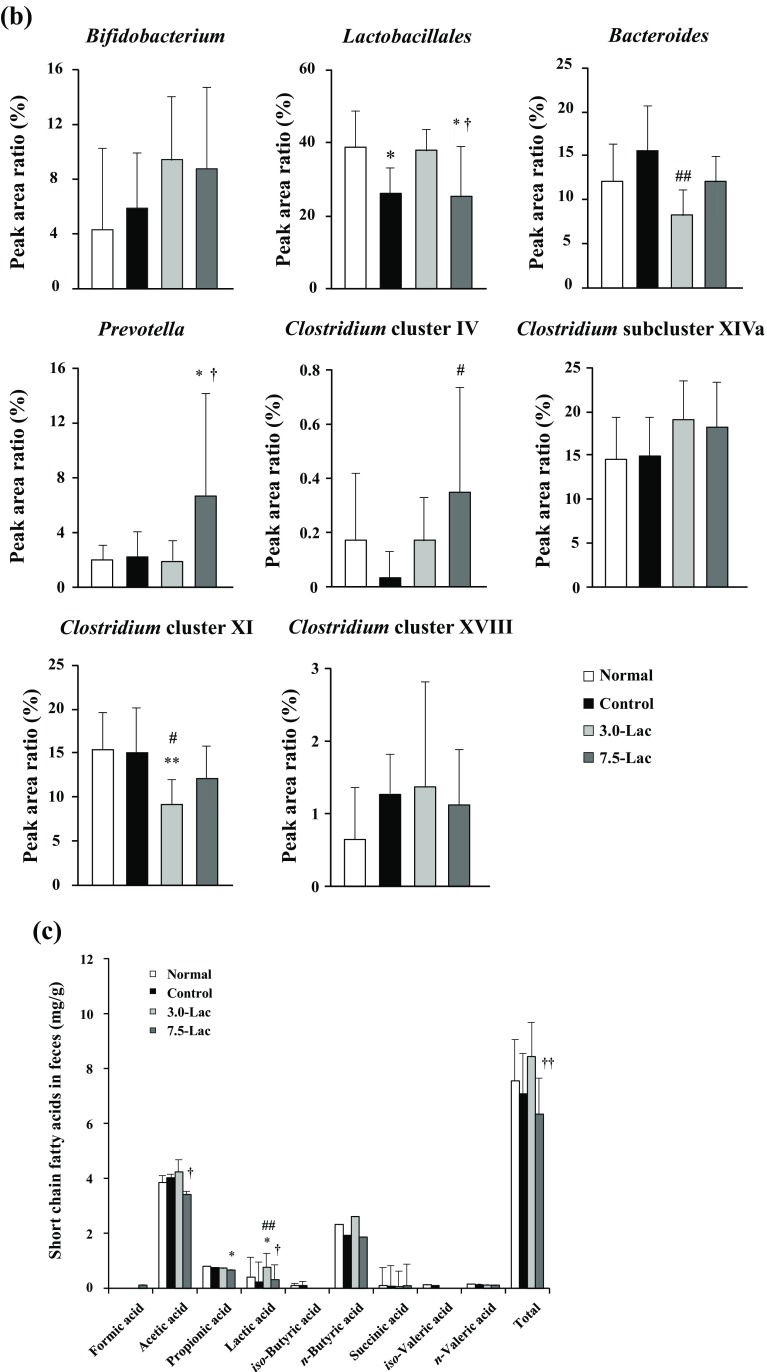
Effects of lactulose on relative abundance of microbiota (**a, b**) and short-chain fatty acid (**c**) in adenine-induced chronic kidney disease (CKD) rats. Alteration of composition of intestinal bacterial flora analyzed using terminal fragment length polymorphism (T-RFLP) analysis. Relative abundance of microbiota based on the average number of each subfamily at the order and genus levels (**a**). Each subfamily is represented in separate graphs (**b**). Concentrations of short-chain fatty acids (**c**) in cecal contents were measured using post-column pH-buffered electroconductivity detection. Values are expressed as mean ± SD; *n* = 10–12/group. *p < 0.05 and **p < 0.01 vs. normal group, ^#^*p* < 0.05 and ^##^*p* < 0.01 vs. control group, and ^†^*p* < 0.05 and ^††^*p* < 0.01 vs. 3.0-Lac group

Peak area ratios of the entire microbiota and individual bacterial taxa at week 4 are compared across the groups in Fig. 7a, b. The *Bacteroides* [13] and *Clostridium* cluster XI [14], which both include many bacterial species producing indole, a precursor of IS, showed decreased peak area ratios in the Lac groups compared with the control group. The *Bifidobacterium* peak area ratio was increased more in the Lac groups than in the control group. Quantitative analysis of gut microbiota using 4′,6-diamidino-2-phenylindole (DAPI) staining did not indicate a difference among the four groups (data not shown).

The quantitative results of short-chain fatty acids in the cecal content at week 4 (Fig. 7c) did not demonstrate a meaningful difference between the control and the normal groups. No apparent changes were induced by the lactulose diet.

## Discussion

The adenine-induced CKD rat was reported by Yokozawa et al. [15] as a renal failure model. Orally ingested adenine is rapidly metabolized to 2,8-dihydroxyadenine, which is deposited and crystallized in the microvilli and the apical domains of the epithelia in proximal renal tubules, causing renal tubule and interstitium degeneration and progression to renal failure [16]. The control rats, which were initially fed a 0.75% adenine-containing diet for 3 weeks and then switched to normal diet for 4 weeks, showed markedly increased sCr and BUN compared with levels before adenine feeding. The rats also exhibited significant tubulointerstitial fibrosis and anemia. These results indicated that the model was a progressed stage of CKD [17].

We also speculated that adenine-induced tubular disorder suppressed renal excretion of uremic toxins [18–20], leading to increase of serum IS, PCS, and TMAO levels even after 4 weeks on normal diet. Additionally, adenine-induced CKD rats are reported to have intestinal permeability increase [21] which can contribute to increase of these uremic toxins as well. Because elevated levels of IS and PCS are known to exacerbate tubular disorder [22, 23], higher IS, and PCS levels in our study likely promoted CKD progression. In contrast, both Lac groups showed decreased levels of serum IS compared with the level before the lactulose diet, and the difference was significant compared with the control group. In the formation of IS, tryptophan from dietary protein is metabolized by enteric bacteria into indole, which is absorbed from the portal vein, conjugated in the liver, and enters the circulation [24]. Based on our observation, the proportion of bacterial taxa containing many indole-producing bacterial species was reduced by lactulose compared with the control group. The decreased serum IS induced by lactulose was likely attributable to suppressed metabolic conversion into indoles in the intestine. Furthermore, the high correlation between serum IS and creatinine or BUN supports that lactulose improved the renal function by reducing serum IS levels. Both Lac groups also exhibited significantly lower serum PCS levels than the control group did. PCS is converted from tyrosine via a similar route to IS [24]. Therefore, the lactulose-induced decrease in serum PCS seemed to also contribute to improving the renal function. Spherical adsorptive carbon used as an oral medication absorbs indole and *p*-cresol in the intestine reduces serum levels of IS and PCS [25], and its efficacy for the treatment of CKD has been demonstrated [26]. Thus, lactulose is expected to be beneficial for patients with CKD, similar to spherical adsorptive carbon.

In addition, IS induces abnormal regulation of oxygen metabolism in renal tubules and suppresses erythropoietin production [27]. The dose-dependent improvement of the red blood cell count, hemoglobin, and hematocrit levels in the lactulose diets suggests the contribution of suppressed erythropoietin production.

IS and PCS are also involved in oxidative stress and renal fibrosis. IS enters the cell via organic anion transporter (OAT) and induces the production of reactive oxygen species (ROS) by NADPH oxidase [28]. ROS stimulate the expression of various cytokines such as TGF-β and induce epithelial-to-mesenchymal transition (EMT), promoting renal fibrosis [29]. PCS also promotes renal disorder by activating NADPH oxidase to generate ROS, which produce fibrosis-related factors including TGF-β and stimulates tubular degeneration and tubulointerstitial fibrosis [30]. In present study, the lactulose-induced decrease in IS and PCS seemed to contribute to suppress tubulointerstitial fibrosis. Moreover, our findings showed that an antioxidant thiol was increased and TGF-β mRNA was slightly decreased. However, further investigations, such as oxidative stress experiments would be needed to clarify the mechanism of lactulose.

In conclusion, our results indicated that lactulose altered the intestinal environment as a prebiotic and, thereby, suppressed uremic toxin production, consequently suppressing the renal disorder and tubulointerstitial fibrosis progression. The present findings are important because modification of the intestinal environment using prebiotics, including lactulose, could be a novel therapeutic approach in preventing CKD progression.
